# Oral Feeding of an Antioxidant Cocktail as a Therapeutic Strategy in a Mouse Model of Rett Syndrome: Merits and Limitations of Long-Term Treatment

**DOI:** 10.3390/antiox11071406

**Published:** 2022-07-20

**Authors:** Laura Baroncelli, Stefanie Auel, Lena Rinne, Ann-Kathrin Schuster, Victoria Brand, Belinda Kempkes, Katharina Dietrich, Michael Müller

**Affiliations:** 1 Institut für Neuro- und Sinnesphysiologie, Universitätsmedizin Göttingen, Georg-August-Universität Göttingen, Humboldtallee 23, D-37073 Göttingen, Germany; laura.baroncelli@in.cnr.it (L.B.); stefanieauel@yahoo.de (S.A.); lena.rinne@gmx.net (L.R.); ak_schuster@web.de (A.-K.S.); victoriabrand@gmx.de (V.B.); bhildeb2@gwdg.de (B.K.); dietrich.katharina85@gmail.com (K.D.); 2 Institute of Neuroscience, National Research Council (CNR), via Giuseppe Moruzzi 1, I-56124 Pisa, Italy; 3 Department of Developmental Neuroscience, IRCCS Stella Maris Foundation, Viale del Tirreno 331, I-56128 Pisa, Italy

**Keywords:** oxidative stress, reactive oxygen species (ROS), disease progression, Mecp2, DNA oxidation, breathing, treatment, mitochondria

## Abstract

Rett syndrome (RTT) is a severe neurodevelopmental disorder that typically arises from spontaneous germline mutations in the X-chromosomal methyl-CpG binding protein 2 (*MECP2*) gene. For the first 6–18 months of life, the development of the mostly female patients appears normal. Subsequently, cognitive impairment, motor disturbances, hand stereotypies, epilepsy, and irregular breathing manifest, with previously learned skills being lost. Early mitochondrial impairment and a systemic oxidative burden are part of the complex pathogenesis, and contribute to disease progression. Accordingly, partial therapeutic merits of redox-stabilizing and antioxidant (AO) treatments were reported in RTT patients and *Mecp2*-mutant mice. Pursuing these findings, we conducted a full preclinical trial on male and female mice to define the therapeutic value of an orally administered AO cocktail composed of vitamin E, N-acetylcysteine, and α-lipoic acid. AO treatment ameliorated some of the microcephaly-related aspects. Moreover, the reduced growth, lowered blood glucose levels, and the hippocampal synaptic plasticity of *Mecp2*^−/*y*^ mice improved. However, the first-time detected intensified oxidative DNA damage in *Mecp2*-mutant cortex persisted. The behavioral performance, breathing regularity, and life expectancy of *Mecp2*-mutant mice did not improve upon AO treatment. Long-term-treated *Mecp2*^+/−^ mice eventually became obese. In conclusion, the AO cocktail ameliorated a subset of symptoms of the complex RTT-related phenotype, thereby further confirming the potential merits of AO-based pharmacotherapies. Yet, it also became evident that long-term AO treatment may lose efficacy and even aggravate the metabolic disturbances in RTT. This emphasizes the importance of a constantly well-balanced redox balance for systemic well-being.

## 1. Introduction

Rett syndrome (RTT) is an X-linked neurodevelopmental disorder primarily affecting females. Typically, a period of 6–18 months of apparently normal development is followed by neurological regression with the loss of acquired cognitive, social, and motor skills [1,2]. Other RTT features include stereotypic hand movements, seizures, ataxia, and cardiac and breathing abnormalities [3,4,5]. At the morphological level, the brain of RTT patients shows an increased cell packing density and a reduction in dendritic arborizations, without neurodegeneration or overt gliosis [6,7]. RTT is caused in the vast majority of cases by spontaneous mutations in the gene encoding the transcriptional regulator methyl CpG binding protein 2 (MeCP2) [8,9,10,11]. No satisfactory treatments are available for RTT, and the mainstay of care is a palliative approach for managing seizures and behavioral problems. Although being relatively rare, RTT represents a major issue in health care, leading to a significant decrease in life expectancy and causing chronic illnesses with a large impact on patients’ quality of life. Therefore, there is a pressing need to identify effective therapies for this devastating disorder [7].

During the last two decades, there has been a dramatic increase in RTT research with the development of mouse models carrying either deletions, duplications, or mutations in the *Mecp2* gene. These mice recapitulate many of the neurological deficits observed in patients, including cognitive, social, and motor impairment, autistic-like stereotypies, and autonomic dysfunctions, such as respiratory dysrhythmia [12,13,14]. The study of animal models demonstrated that MeCP2 is essential in the modulation of synaptic functions during early postnatal development, indicating that RTT results from the functional disruption of synaptic plasticity and neuronal circuits [3,15], with a significant imbalance between excitation and inhibition [16]. Despite the continuing progress in the knowledge of RTT natural history, the deciphering of MeCP2 function, and the mapping of circuit-specific alterations in *Mecp2*-deficient brains, the molecular mechanisms leading from the defective protein to the disease manifestation are not fully understood. This type of detailed information is crucial, however, to uncover possible druggable targets.

Oxidative stress plays an important role in the pathogenesis and the progression of RTT [17,18,19]. It is accompanied by disturbed redox homeostasis, mitochondrial alterations [17,20,21,22,23,24,25], impairment of antioxidant defense [26,27,28], and increased systemic oxidative damage to proteins and lipids [27,29,30]. The fibroblasts and neural circuits of RTT patients also show signs predictive of oxidative damage [31,32,33]. Importantly, oxidative stress is a critical feature in the brain of *Mecp2*-mutant mice and RTT patients even before the appearance of typical symptoms [23,30,34,35]. Moreover, the improvement of the endophenotype induced in RTT mouse models by the re-expression of the *Mecp2* gene is accompanied by a significant reduction in brain oxidative-stress-marker levels [30]. According to this vision, treatments based on free-radical scavengers and/or antioxidants (AOs) showed some merits, but also certain limitations in mouse models of RTT [36,37,38,39] as well as in patients [40,41,42]. However, a systematic and long-term evaluation of AO-based therapies is still missing.

Here, we tested the efficacy of early targeted drug therapy using a cocktail of AOs that was previously reported to mediate neuroprotection against the degeneration of spinal axons and to prevent pro-inflammatory alterations in the aging brain [43,44]. This AO cocktail was composed of vitamin E, α-lipoic acid, and N-acetyl cysteine, and it was administered as customized food pellets right from the time point of weaning. In detail, we defined its impact on phenotypic alterations, and on morphological, biochemical, and functional parameters at various stages of the RTT disorder in male as well as female mice. Furthermore, we assessed in female mice the effects of long-term AO treatment by monitoring them up to postnatal day (pd) 400.

## 2. Materials and Methods

### 2.1. Animals and Genotyping

In accordance with our previous studies, we used the *Mecp2* knock-out mouse model for RTT (B6.129P2(C)-Mecp2tm-1-1Bird [45]). For stable colony breeding, fresh heterozygous female mice were occasionally obtained from Jackson Laboratories (Bar Harbor, ME, USA). These female mice were bred at the central animal facility of University Medical Center Göttingen with male wildtype (WT) mice (C57BL/6J), to obtain hemizygous males (*Mecp2*^−/*y*^), heterozygous females (*Mecp2*^+/−^), and WT mice of both genders.

### 2.2. Systemic AO Treatment

The systemic AO treatment of mice was realized with a special diet obtained from SSNIFF Spezialdiäten GmbH (Soest, Germany). It was based on the regular mouse diet (V1124-0, SSNIFF), supplemented with 250 mg/kg of diet α-lipoic acid, 2.5 g/kg of diet N-acetylcysteine, and an additional 125 mg/kg of diet vitamin E (α-tocopherol). Food was provided *ad libitum*, and pharmacotherapy was based on self-feeding. Assuming that a mouse consumes 4 g of food per day [46], we calculated, based on a body weight of 25 g, a daily AO dose of 40 mg/kg/day of α-lipoic acid, 400 mg/kg/day of N-acetyl cysteine, and 40 mg/kg/day of vitamin E. Entire mouse litters were randomly assigned to either normal or AO food. The respective treatment started right upon weaning (~pd 20) and was continued until the time point of analysis. Mice were closely monitored, and in case of complications, i.e., obvious signs of suffering or pain, difficulty in breathing, massive weight loss, lethargy, tremors, or ruffled fur, the treatment was terminated, and the given mouse was euthanized.

### 2.3. Behavioral Testing

To define the phenotypic conditions of mice, general physiological parameters (body weight, body size, hematocrit, blood glucose level) were determined. Moreover, the various cohorts of mice underwent different behavioral paradigms to rate motor coordination and environmental exploration. Behavioral tests were conducted for both genders on pd 50, and they were repeated on pd 200 for female mice. A subset of mice served to define total lifespans.

Rotarod testing revealed the motor coordination skills. Mice were individually placed on a drum; the rotation speed was increased from 5 to 50 rpm, and the total time spent on the drum (maximum speed reached after 5 min) was recorded. Motor abilities were assessed by conducting the test on three consecutive days. If the mice just clasped to the drum instead of running, the experiment was stopped as soon as 5 full rotations of clasping were completed [37].

The hole-board test assessed motor activity and environmental exploration. Mice were placed in the center of the open arena (45 × 45 cm; 16 equally spaced holes of 25 mm diameter in the floor), and their motility was tracked over 5 min by a grid of infrared beams (ActiTrack v2.7.13 software; Panlab, Harvard Apparatus). For position analyses, the arena was divided into 3 virtual zones: center, periphery, and corners. In addition, the exploration of the holes in the base plate was recorded as the total number of head dips [37].

### 2.4. Plethysmography

Breathing was rated using a whole-body plethysmograph. The mice were placed in a plethysmographic chamber (Data Sciences International (DSI) St. Paul, MN, USA; Ponemah v5 software), and their respiration behavior was monitored under conscious, unrestrained conditions for 10 min. To allow them to adapt to the recording situation, only the last 3 min of the total recording time were analyzed. Occasionally, plethysmography was combined with video-recordings of the respective mouse, to correlate their complex breathing patterns with the displayed behavior. For respiration analyses, the individual respiratory cycles were detected using threshold-crossing-based detection (Clampfit 10.3; Molecular Devices). Based on these data, the instantaneous breathing frequencies were calculated. In addition, the irregularity score was determined as the normalized difference between two subsequent breaths [37,47].

### 2.5. Brain-Tissue Isolation

To isolate fresh brain tissue at the desired ages, the respective mouse was deeply ether-anesthetized and decapitated. The brains were rapidly but gently removed from the skull and placed in chilled phosphate-buffered saline (PBS) for 1–2 min. The desired brain regions were then separated and flash-frozen in liquid nitrogen and cryopreserved at −80 °C for later biochemical analyses (DNA oxidation). Alternatively, acute cortico-hippocampal tissue slices (400 µm thickness) were prepared for the electrophysiological recordings, following our previously described procedures [38]. The slices obtained were cut along the sagittal midline, and the resulting hemislices were kept submerged at room temperature in a storage chamber for at least 90 min before the electrophysiological recordings were started.

To provide properly fixed brain tissue for the morphometric analyses, a subset of mice underwent transcardial perfusion [37]. A sufficiently deep ether anesthesia was verified by the absence of foot-pinch reflexes. The heart was exposed by opening the chest; then, an injection cannula was carefully inserted into the left ventricle, and by perfusion with PBS, any remaining blood was washed out from the vascular system. Then, the perfusate was switched to 4% paraformaldehyde (PFA) in PBS. After ~5 min of PFA perfusion, the brain was isolated, postfixed for the next 2–3 days in 4% PFA, and then stored until further processing in PBS at 4 °C.

### 2.6. Chemicals and Solutions

Unless explicitly differently stated, all drugs and compounds were purchased from Sigma-Aldrich (St. Louis, MO, USA). PBS was made of 137 mM NaCl, 2.7 mM KCl, 10 mM Na_2_HPO_4_, and 2 mM KH_2_PO_4_; a pH of 7.4 was adjusted via titration with 1 M NaOH. ACSF (artificial cerebrospinal fluid) contained 130 mM NaCl, 24 mM NaHCO_3_, 3.5 mM KCl, 1.2 mM CaCl_2_, 1.2 mM MgSO_4_, 1.25 mM NaH_2_PO_4_, and 10 mM dextrose; a pH of 7.4 was adjusted via constant aeration with carbogen (95% O_2_, 5% CO_2_).

### 2.7. Nissl Staining of Brain Slices

The fixed brains were sliced as coronal sections (30 µm), by using a vibrating microtome (VT1200S, Leica Biosystems, Nußloch, Germany). The desired sections were then placed on microscope slides and dried for 30–60 min before they were passed through a descending ethanol series (95% for 15 min; 70% and 50% for 1 min each) and finally washed twice in distilled water. Next, the microscope slides with the mounted sections were stained for 2 min in an aqueous cresyl violet/acetate solution (5 mg/mL cresyl violet, containing 30 µL/mL acetic acid). The remaining dye was washed off with distilled water, and sections were passed through a series of ascending EtOH dilutions (50% for 1 min, 70% plus 1% acetic acid for 2 min, 95% for 2 min; 99.9% for 2 min). Finally, the sections were cleared via xylol incubation (5 min), dried for 15 min, and coverslipped with a quick-hardening mounting medium (Eukitt, ORSAtec, Bobingen, Germany). Images of the tissue sections were taken with a digital microscope (Coolscope, Nikon, Minato, Japan), and NIS-Elements 3.22 cell-analysis software was used for all morphometric measurements. Total hemisphere size and cortical-layer thickness were quantified for the left and right hemisphere and averaged. The size of single neurons (soma area) was determined from 5 randomly chosen cells of each hemisphere and then averaged for the given tissue section. For these analyses, we selected tissue sections from a rostro-caudal position of about −1.7 mm with respect to Bregma [37,48].

### 2.8. Electrophysiological Recordings

Synaptic short-term potentiation (STP) and long-term potentiation (LTP) of the hippocampal network were analyzed in acute brain tissue slices of the pd 50 male-mouse groups. Recordings were conducted in an Oslo-style interface recording chamber, which was operated at a temperature of 31–32 °C, was aerated continuously with humidified carbogen (400 mL/min), and was perfused with oxygenated ACSF (3–4 mL/min). Extracellular recording electrodes were pulled from thin-walled borosilicate glass capillaries (GC150TF-10; Harvard Apparatus, Holliston, MA, USA) using a horizontal electrode puller (Model P-97; Sutter Instruments, Novato, CA, USA). Electrodes were filled with ACSF, and their tips were trimmed to a resistance of ~5 MΩ. Field excitatory postsynaptic potentials (fEPSPs) were elicited by 0.1 ms unipolar stimuli (S88 stimulator with PSIU6 stimulus-isolation units; Grass Instruments, West Warwick RI, USA) using steel microwire electrodes (50 µm in diameter; AM-Systems, Sequim, WA, USA) placed in the Schaffer collaterals. Orthodromic responses were recorded in the *st. radiatum* of the hippocampal CA1 subfield using a locally constructed extracellular DC-potential amplifier, Axon Instruments Digitizer 1322A, and the PClamp 9.2 software package (Molecular Devices, San Jose CA, USA) [49]. The sampling rate was set to 20 kHz; stimulus intensities were adjusted to yield half-maximum fEPSP amplitudes, and 4 consecutive fEPSPs (evoked every 15 s) were averaged. LTP was induced in ACSF, i.e., under the conditions of normal extracellular Ca^2+^ concentrations (1.2 mM), by applying three trains of 100 Hz stimuli (1 s train duration, separated by 5 min each) [38]. STP was rated immediately after the 3rd stimulus train (post-tetanic potentiation (PTP)), whereas LTP was assessed in the time window of 50–60 min after high-frequency stimulation.

### 2.9. DNA-Oxidation Assay

To quantify the extent of DNA oxidation, the cortex was isolated from male/female WT and *Mecp2*-mutant mice, flash-frozen in liquid nitrogen, and cryopreserved at −80 °C until use. For DNA isolation, entire cortical hemispheres (typically 70–100 mg) were lysed at 55 °C, and cell debris was removed by centrifugation; then, the DNA was precipitated with isopropanol, spun down, and re-dissolved in ultrapure water. Subsequently, the DNA was extracted using QIAmp Fast DNA Tissue Kit (#51404; Qiagen, Hilden, Germany). The extent of DNA oxidation was assessed by measuring the concentration of oxidized guanine species (8-OHdG) with a spectrophotometric assay (DNA/RNA oxidative damage ELISA Kit; #589320; Cayman Chemicals, Ann Arbor, MI, USA), following the manufacturer’s instructions. The 96-well plates were read at a 412 nm absorption using a multi-mode spectrofluorometer (flx-Xenius; SAFAS Monaco, Monaco).

### 2.10. Statistics

For the *in vitro* analyses, up to 3 slices or tissue samples were used from each brain. These were considered technical repeats and were averaged for the respective mouse. To ensure the independence of observations, each series of experiments included at least 5 different mice of each experimental group. All numerical values are represented as means ± standard deviations. The number of experiments (*n*) refers to the number of mice analyzed. For the direct comparison of two groups, the statistical significance of the differences and changes observed was tested by two-tailed unpaired Student’s *t*-tests. In the case of normalized data, one-sample *t*-tests were used to compare the differences from unity (1.0 or 100%). In the case of multiple-group comparisons, two-way ANOVAs followed by Holm–Sidak all-pairwise-comparison *post-hoc* tests were conducted. The lifetime of the different mice was determined with a Kaplan–Meier survival analysis (log-rank test followed by a Holm–Sidak *post-hoc* test). All statistical calculations were computed using SigmaStat 3.5 (Systat Software, Erkrath, Germany). In the diagrams, statistically significant changes between genotypes are indicated by asterisks (* *p* < 0.05; ** *p* < 0.01). Treatment-related differences in either WT or *Mecp2*-mutant mice are identified by crosshatches (# *p* < 0.05, ## *p* < 0.01).

## 3. Results

To define the detailed therapeutic merit of a systemic treatment of *Mecp2*-mutant mice with the AO cocktail, we assessed multiple systemic, behavioral, morphometric, electrophysiological, and biochemical parameters in male and female mice. Both genders of wild-type (WT) and *Mecp2*-mutant mice were analyzed on postnatal day (pd) 50. In addition, female mice were studied on pd 200 and on pd 400 to assess the outcome of long-term AO treatment.

### 3.1. General Phenotypic Appearance and Blood Parameters

**Figure 1:**
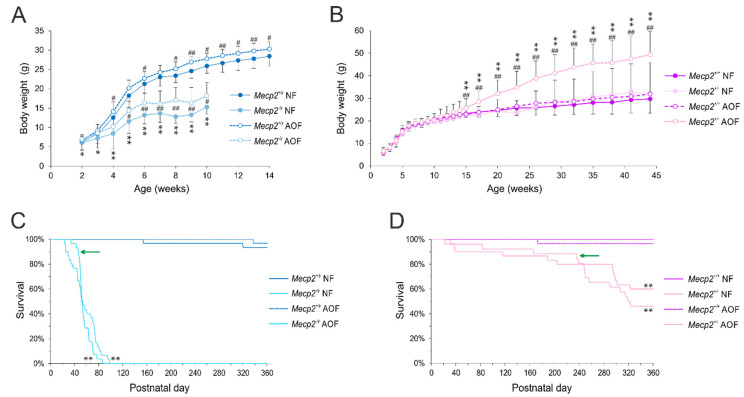
Antioxidant food (AOF)-mediated modulation of body weights and life expectancy. (**A**,**B**) In *Mecp2*^−/*y*^ mice, the AOs improved the weight gain but evoked an obese phenotype in *Mecp2*^+/−^ mice. Plotted are the mean body weights ± standard deviations of 22–38 mice per group. Significant body-weight changes as compared with the respective WT group (two-way ANOVA, followed by Holm–Sidak *post-hoc* test) are indicated by asterisks (* *p* < 0.05, ** *p* < 0.01), and AO-treatment-mediated effects among either WT or *Mecp2*-mutant mice are identified by crosshatches (# *p* < 0.05, ## *p* < 0.01). (**C**,**D**) In the Kaplan–Meier survival curves for male and female mice, the total life expectancy was not prolonged by AO treatment, but in *Mecp2*^−/*y*^ and *Mecp2*^+/−^ mice, premature death during the first half of life tended to be less frequent (see green arrow marks). Plotted curves are based on 26–30 mice per group. Significant changes in the lifetime as compared with WT conditions with normal food (NF) were assessed using log-rank tests followed by Holm–Sidak pairwise comparisons (** *p* < 0.01).

As *Mecp2*^−/*y*^ mice typically show a reduced growth, we closely monitored their body weight during development and adulthood. As expected, the weight gain of *Mecp2*^−/*y*^ mice was clearly reduced as compared with WT mice, resulting at 8 weeks of age in a 49% lower body weight than that of WT males (Figure 1A). In contrast, *Mecp2*^+/−^ mice did not differ in their weights from female WT mice (Figure 1B). AO food successfully improved the growth of *Mecp2*^−/*y*^ mice, resulting in 34% higher body weights at 8 weeks of age than those of *Mecp2*^−/*y*^ mice receiving normal food. In addition, AO-fed WT males gained slightly more weight than those fed regular food (Figure 1A). In *Mecp2*^+/−^ mice, however, AO treatment caused a massive weight gain, which started from 15 weeks of age. In particular, during the continued long-term treatment, the body weights of AO-fed *Mecp2*^+/−^ mice markedly increased as compared with the other groups, resulting in 60% higher weights at 42 weeks of age (Figure 1B).

The reduced growth of *Mecp2*^−/*y*^ mice was also evident as a smaller body size and a lower body mass index (BMI) than those of WT males. AO food also improved these parameters (Table 1). In line with the massive increase in body weight, AO-treated *Mecp2*^+/−^ females showed a markedly higher BMI on pd 400.

Previously, we reported an increased hematocrit and a reduced blood glucose level in symptomatic *Mecp2*^−/*y*^ mice [37,50]. This also applied to the current mouse cohort, and an increased hematocrit was also evident in *Mecp2*^+/−^ females (Table 1). AO treatment did not counteract the increased hematocrit in *Mecp2*^−/*y*^ mice and even tended to further increase it in *Mecp2*^+/−^ females (Table 1). The blood glucose levels showed heterogeneous changes, being markedly lowered in *Mecp2*^−/*y*^ males but tending to increase in *Mecp2*^+/−^ females. With AO treatment, the blood glucose level tended to increase in *Mecp2*^−/*y*^ males (Table 1). In old *Mecp2*^+/−^ females (pd 400), a marked further rise in the blood glucose content occurred, which may be linked to the above-mentioned obese phenotype manifesting during long-term AO treatment.

The life expectancy of male and female *Mecp2*-mutant mice was significantly shortened as compared with WT mice—especially in severely affected *Mecp2*^−/*y*^ mice (Figure 1C,D), which did not survive beyond pd 100. AO treatment hardly modulated the life expectancy of *Mecp2*-mutant mice (Figure 1C,D). Total life expectancy remained unchanged in *Mecp2*^−/*y*^ and *Mecp2*^+*/*−^ mice, but AO food tended to reduce the fraction of premature deaths during the first half of life in male and female *Mecp2*-mutant mice. The level of significance was, however, not reached, and once the decline started, it progressed even faster. In the WT groups, any obvious effects of the AO treatment on life expectancy did not occur (Figure 1C,D).

### 3.2. Behavioral Assessment

**Figure 2:**
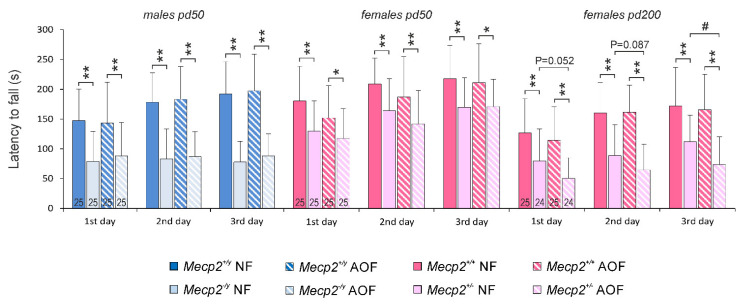
Rotarod testing revealed poor motor coordination in *Mecp2*-mutant mice but no beneficial effects of AO treatment. Plotted are the average durations the mice stayed on the rod. The test was conducted on 3 consecutive days. While the performance of WT mice improved over the 3 days, no such improvement was seen in *Mecp2*^−/*y*^ mice. On pd 200, the long-term AO-treated *Mecp2*^+/−^ mice performed even worse than untreated *Mecp2*^+/−^ mice. Significant differences were assessed by two-way ANOVAs and Holm–Sidak *post-hoc* tests and are indicated by asterisks (* *p* < 0.05, ** *p* < 0.01). Treatment-related differences among *Mecp2*^+/−^ mice are indicated by crosshatches (# *p* < 0.05).

The rotarod test revealed the expected impaired motor dysfunction in *Mecp2*^−/*y*^ mice, which was apparent as clearly shorter times spent on the rotarod, without any improvements along the 3 days of testing. A corresponding motor impairment was evident in heterozygous females on pd 50 and pd 200, although *Mecp2*^+/−^ females did improve their performance during the test (Figure 2). The AO-enriched diet did not ameliorate the motor performance of *Mecp2*^−/*y*^ and *Mecp2*^+/−^ mice on pd 50. With continued AO treatment, *Mecp2*^+/−^ mice even developed a further decline in motor performance on pd 200. In the WT groups, any significant effects of AO food could not be detected. Note, however, that AO-fed female WT mice tended to show a weaker performance on pd 50 (Figure 2).

**Figure 3:**
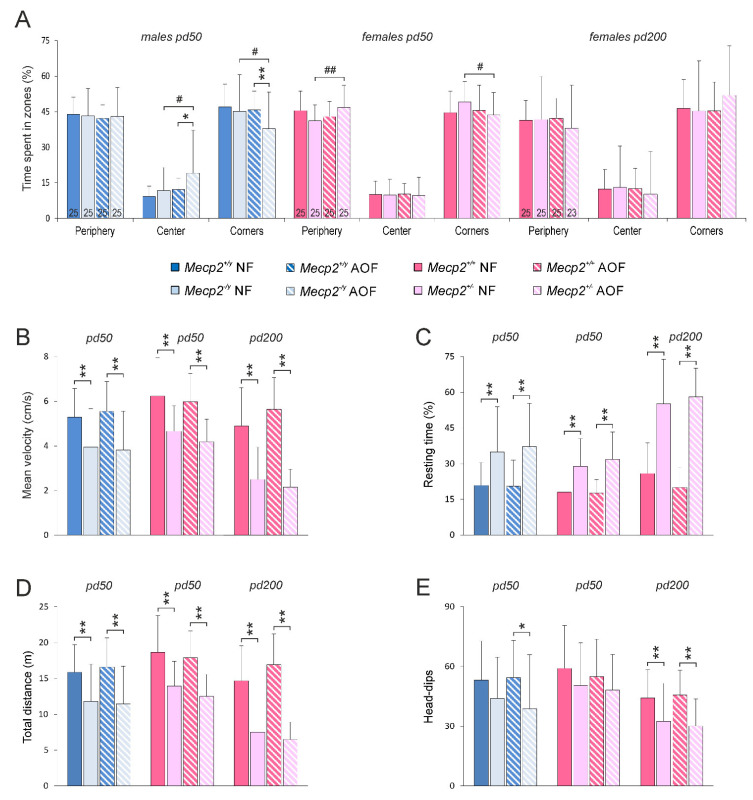
General activity and environmental exploration were assessed by hole-board testing. (**A**) Mice preferred the corners and peripheral sections of the open area. Marked genotype- or treatment-related differences in these preferences were not evident. (**B**) Male and female *Mecp2*-mutant mice were less active than their WT siblings and moved at lower velocities. (**C**,**D**) Furthermore, they rested longer than the respective WT mice and ran shorter total distances. (**E**) The environmental exploration, indicated here as head-dips into the holes of the base plate, was also less pronounced. Neither in male nor female mice did AO treatment improve any of these parameters. Plotted are means ± standard deviations, and 23–25 mice per group were studied, as indicated in panel (**A**). Mouse number, bar shading, and colors apply to all panels. Significant differences were confirmed by two-way ANOVAs followed by Holm–Sidak tests. Genotype-related differences are marked by asterisks (* *p* < 0.05, ** *p* < 0.01); AO-treatment-related differences are identified by crosshatches (# *p* < 0.05, ## *p* < 0.01).

The hole-board paradigm indicated for all mice a clear preference for the peripheral areas and the corners of the arena, as compared with the open center. These spatial preferences did not differ among normally fed WT and *Mecp2-mutant* mice, nor were there any marked changes induced by AO treatment (Figure 3A). Nevertheless, *Mecp2*^−/*y*^ mice showed a markedly poorer performance in the hole-board arena than their WT siblings. They moved at lower velocities (Figure 3B), rested longer (Figure 3C), ran shorter total distances (Figure 3D), and tended to explore fewer holes of the base plate (number of head-dips) than WT males (Figure 3E). Similar patterns of reduced activity, less intense exploration, shorter distances, and increased resting times were observed in *Mecp2*^+/−^ mice on pd 50, and this became even more pronounced on pd 200. Treatment with AOs did not improve any of the parameters of the hole-board test, neither in *Mecp2*^−/*y*^ males nor in the two age groups of *Mecp2*^+/−^ females. Any negative effects of AO treatment in the WT groups were not observed (Figure 3A–E).

### 3.3. Whole-Body Plethysmography

**Figure 4:**
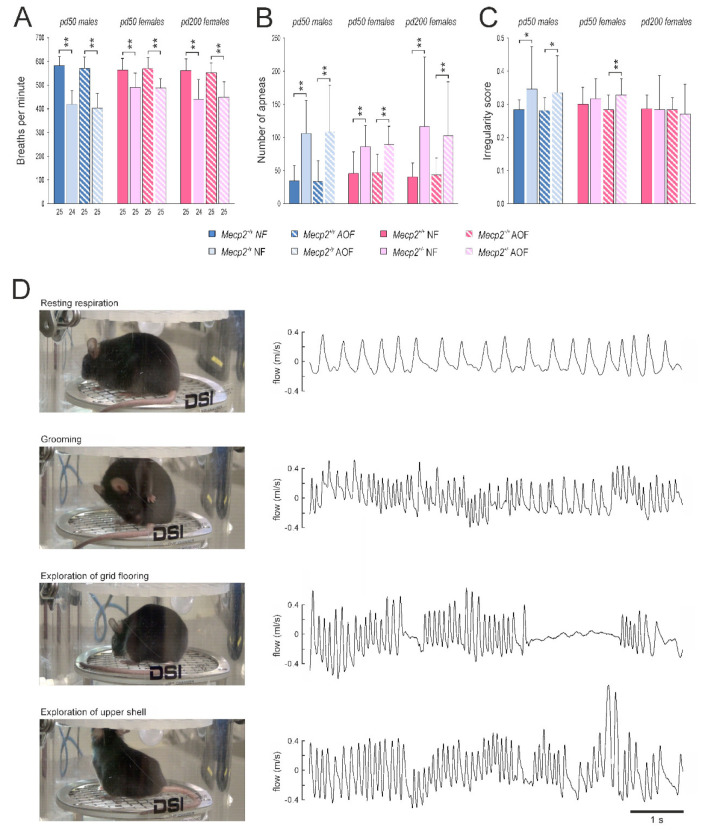
The distorted breathing of *Mecp2*-mutant mice was not rescued by AO treatment. (**A**) Both *Mecp2*^−/*y*^ and *Mecp2*^+/−^ mice showed markedly higher breathing frequencies than the respective WTs. (**B**) Breathing arrests (apneas exceeding 250 ms duration; counted over 3 min) were far more frequent in male and female *Mecp2*-mutant mice. (**C**) Moreover, the regularity of breathing was/tended to be more distorted in *Mecp2*^−/*y*^ and *Mecp2*^+/−^ mice on pd 50 than in WT mice. None of these respiratory parameters were affected by the AO cocktail. (**D**) Unrestrained whole-body plethysmography gave rise to a complex breathing behavior, which may markedly differ from resting respiration. Especially, exploratory activity and grooming were associated with more irregular breathing patterns and higher frequencies. Pictures and traces were obtained from an AO-fed WT female on pd 200. The number of mice analyzed is indicated in panel (**A**). Genotype-related significant changes are identified by asterisks (* *p* < 0.05, ** *p* < 0.01; two-way ANOVAs followed by Holm–Sidak *post-hoc* tests).

The respiration behavior of male and female mice was analyzed on pd 50 and for females also on pd 200. As main parameters we assessed the breathing frequency (breaths per minute (BPM)), the interevent duration between two breaths, and the irregularity score. Furthermore, the number of apneas was counted. As expected, *Mecp2*^−/*y*^ mice showed markedly (~28%) lower breathing frequencies than WT males (Figure 4A). A frequency reduction (by 13%) was seen in *Mecp2*^+/−^ mice, even though they were considered to be mostly pre-symptomatic on pd 50. At a more progressed disease state (pd 200), the frequency reduction in *Mecp2*^+/−^ mice became more apparent (~21% as compared with WT; Figure 4A). In none of the groups, AO-treatment mediated noticeable changes in the respiration frequencies, nor did it ameliorate the RTT-related reduction in breathing frequency (Figure 4A).

Another hallmark of the breathing patterns in *Mecp2*-mutant mice was a significantly higher number of apneas (breathing arrests), which could be detected in *Mecp2*^−/*y*^ as well as *Mecp2*^+/−^ mice. Counting the number of apneas with a duration of more than 250 ms revealed a threefold occurrence in *Mecp2*^−/*y*^ mice and a twofold prevalence of apneas in *Mecp2*^+/−^ mice as compared with the respective WT groups on pd 50. With aging (pd 200), apneas became somewhat more frequent in *Mecp2*^+/−^ mice (Figure 4B). AO food did not ameliorate these conditions.

As a consequence of the altered respiratory patterns in *Mecp2*-mutant mice, the irregularity scores in *Mecp2*^−/*y*^ mice were increased, and a similar trend was seen in *Mecp2*^+/−^ mice on pd 50 (Figure 4C). On pd 200, any differences were no longer evident among *Mecp2*^+/−^ and WT females. An amelioration of the irregularity scores was not mediated by AO treatment (Figure 4C).

Our respiratory analyses were performed in a whole-body plethysmograph under unrestrained conditions. The mice were able to move around freely in the plethysmographic chamber, to groom, and to explore the chamber (floor grid, lid of the chamber). As documented by video-monitoring, this complex behavior was associated with distinct respiratory patterns (Figure 4D). As long as the mice rested, they presented a rather regular and low-frequency respiration. In contrast, grooming was associated with markedly higher respiration frequencies. The exploration of the lid or the base grid of the chamber was associated with high-frequency sniffing episodes with intermittent episodes of breath holding—in particular during the exploration of the grid flooring (Figure 4D). Plethysmographic analyses revealed that the exploratory behavior and the grooming were associated with respiratory frequencies in the range of 550–700 breaths per minute (BPM).

**Figure 5:**
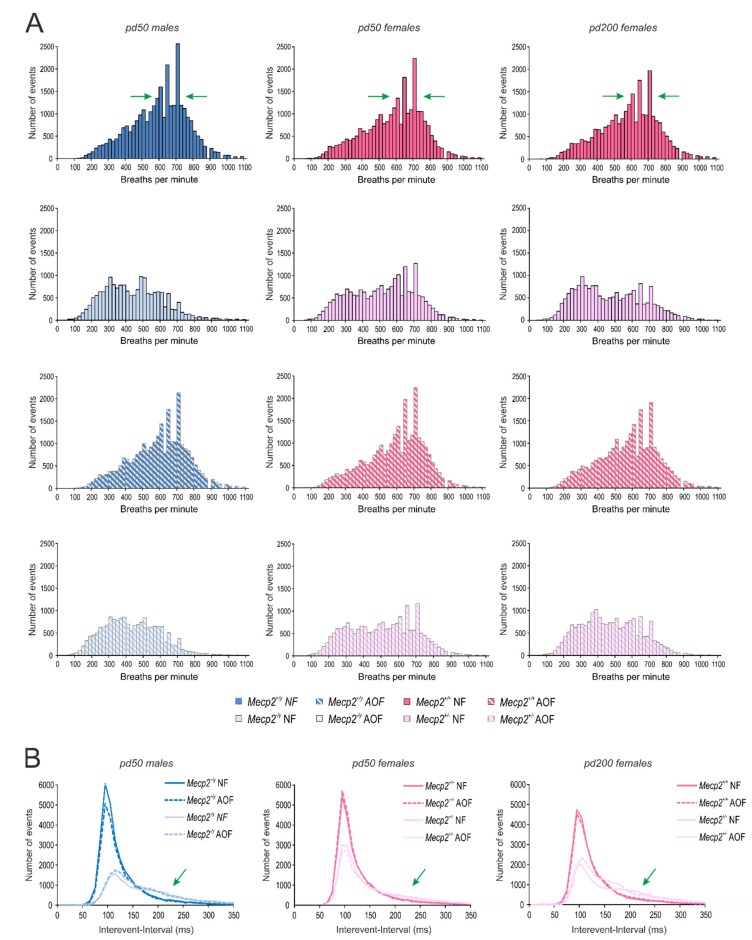
Spectral analyses revealed the full complexity of breathing. (**A**) Breathing patterns were composed of frequencies ranging between 100 and 1100 breaths per minute. In *Mecp2*^−/*y*^ and *Mecp2*^+/−^ mice, this distribution was left-shifted as higher frequencies were less prominent. Green arrows flank those frequencies (550–700 BPM) associated with grooming and exploratory activities. AO treatment did not affect the frequency composition of the respiratory patterns. (**B**) The interevent interval, i.e., the timespan between two breaths, also showed a defined spectral range that was markedly broadened (see arrow mark) in *Mecp2*^−/*y*^ and *Mecp2*^+/−^ mice as compared with WTs. Data represent 24–25 mice per group.

To account for this complexity of breathing, the spectral composition of the plethysmographic traces was plotted as frequency histograms (Figure 5A). The entity of frequencies detected ranged between 100 and 1100 breaths per minute (BPM). As grooming and exploratory activities were observed in particular in WT mice, a marked increase in the respective frequency range (550–700 BPM) was evident in the respective histograms (Figure 5A, see green arrows). In contrast, *Mecp2*^−/*y*^ and *Mecp2*^+/−^ mice presented left-shifted histograms with a lower contribution of higher frequencies. The AO diet did not affect the frequency distribution of the breathing behavior in WT mice, nor did it ameliorate the RTT-related alterations in frequency composition (Figure 5A). Therefore, we performed a corresponding spectral analysis of the interevent intervals, as the heterogeneity of these intervals was one of the main sources of the irregular breathing of *Mecp2*-mutant mice.

In WT mice, the time span between two consecutive breaths was quite homogenous, being evident as a clear peak at 100 ms in the interevent histograms (Figure 5B). In contrast, male and female *Mecp2*-mutant mice showed a more heterogeneous distribution of interevent intervals. Accordingly, the peak at 100 ms was dampened, and a general broadening of the respective histograms towards longer interevent durations was evident (see green arrow marks in Figure 5B). AO treatment did not abolish these RTT-related differences. Only on pd 200, *Mecp2*^+/−^ mice receiving AO food showed a moderate shift of their interevent durations towards shorter intervals (Figure 5B), which might be interpreted as a trend towards shorter apneas.

### 3.4. Brain Morphometry

**Figure 6:**
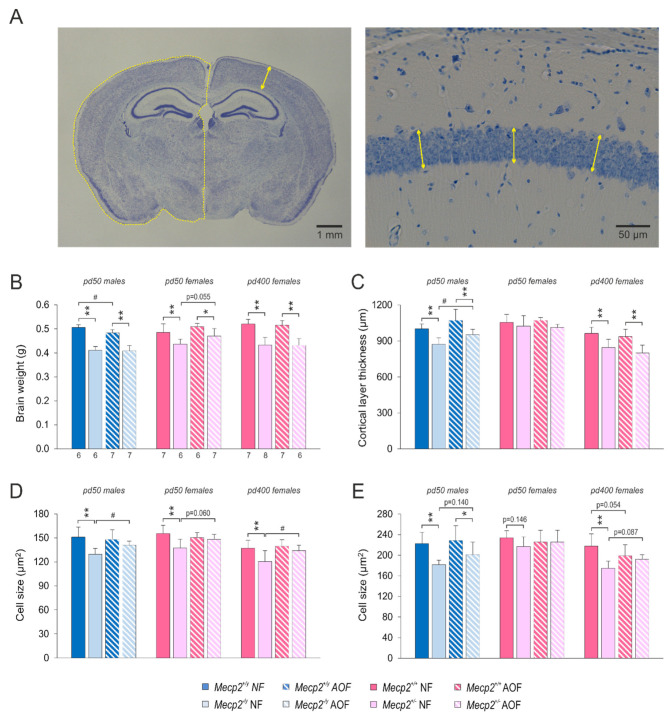
Different aspects of brain morphology showed a partial rescue by AO treatment. (**A**) Nissl-stained tissue sections were obtained from a rostro-caudal position of −1.7 mm with respect to Bregma to quantify hemisphere size, cortical-layer thickness, and the size of individual neurons. Displayed is a section of an AO-treated WT male and a close-up view of CA1 *str. pyramidale* with an indication of the performed measurements. (**B**) Total brain weight was decreased in all *Mecp2*-mutant mice, but AO food only tended to rescue the brain weight of female *Mecp2*^+/−^ mice on pd 50. Plotted weights refer to perfusion-fixed brains (4% PFA), and the number of mice studied is reported. Asterisks indicate statistically significant differences as compared with the respective WT groups (* *p* < 0.5, ** *p* < 0.01). AO-treatment-mediated differences among either WT or *Mecp2*-mutant mice are indicated by crosshatches (# *p* < 0.05). All patterns also apply to the other panels. (**C**) The cortex was thinner in male and pd 400 female *Mecp2*-mutant mice, but total layer thickness was only improved by AO treatment in male *Mecp2*^−/*y*^ mice. (**D**) The CA1 pyramidal neurons were smaller in male and female *Mecp2*-mutant mice, and their size was rescued or tended to be rescued by the AO cocktail. (**E**) The CA3 pyramidal neurons were smaller in *Mecp2*^−/*y*^ mice and in *Mecp2*^+/−^ mice on pd 400, but these differences were not restored by AO treatment.

Among the cardinal features of RTT is microcephaly. Therefore, we assessed various aspects of brain morphometry in Nissl-stained tissue sections (Table 2, Figure 6A). In *Mecp2*^−/*y*^ mice (pd 50) lower brain weights were detected than in WTs. This also applied to *Mecp2*^+/−^ mice on pd 50 and on pd 400 (Figure 6B). The AO diet did not improve the brain weight of hemizygous males, but it dampened the genotypic differences by tending to increase the brain weight in *Mecp2*^+/−^ mice on pd 50 (*p* = 0.055). On pd 400, however, this protection was no longer evident in AO-treated *Mecp2*^+/−^ mice (Figure 6B). Furthermore, the total hemisphere size was reduced in all *Mecp2*-mutant mice (Table 2), and a trend towards partial rescue by AO food was observed in *Mecp2*^+/−^ mice on pd 50 (*p* = 0.135). Moreover, the total cortical-layer thickness was thinner in *Mecp2*^−/*y*^ mice as well as in *Mecp2*^+/−^ mice on pd 400. A rescue of this parameter was only seen in AO-treated *Mecp2*^−/*y*^ mice (Figure 6C).

A closer look was taken at the hippocampal formation, where changes in the width of the different cell layers became evident (Table 2). The pyramidal cell layers (*str. pyramidale*) of the CA1 and CA3 subfields were narrowed in *Mecp2*^+/−^ mice, and the CA1-pyramidal-cell layer tended to be narrowed in *Mecp2*^−/*y*^ mice (*p* = 0.062). The granule-cell layer of the dentate gyrus only showed a similar tendency in *Mecp2*^+/−^ mice on pd 400 (*p* = 0.094). AO treatment partly ameliorated these differences in the *Mecp2*^+/−^ mice. As a result, the CA3-pyramidal-cell layer no longer significantly differed from WT conditions on pd 50, and the CA1- and CA3-pyramidal-cell layers no longer significantly differed from pd 400 WTs. For the granule-cell layer (*str. granulosum*) of dentate gyrus similar trends were observed in *Mecp2*^+/−^ mice; none of them did, however, reach the level of significance (Table 2). The size of CA1 pyramidal neurons was decreased in all *Mecp2*-mutant mice as compared with WT controls. In *Mecp2*^−/*y*^ as well as *Mecp2*^+/−^ mice these genotypic differences were abolished by AO treatment (Figure 6D). In addition, CA3 pyramidal neurons were significantly smaller in *Mecp2*^−/*y*^ mice as well as in *Mecp2*^+/−^ mice on pd 400, and these disease-related differences tended to become less pronounced upon AO-feeding (Figure 6E).

### 3.5. Synaptic Plasticity

To define the genotype- and treatment-related differences in synaptic plasticity, we quantified the extents of hippocampal short-term potentiation (analyzed here as post-tetanic potentiation (PTP)) and long-term potentiation (LTP) at the Schaffer collateral/CA1 synapse of male mice on pd 50.

**Figure 7:**
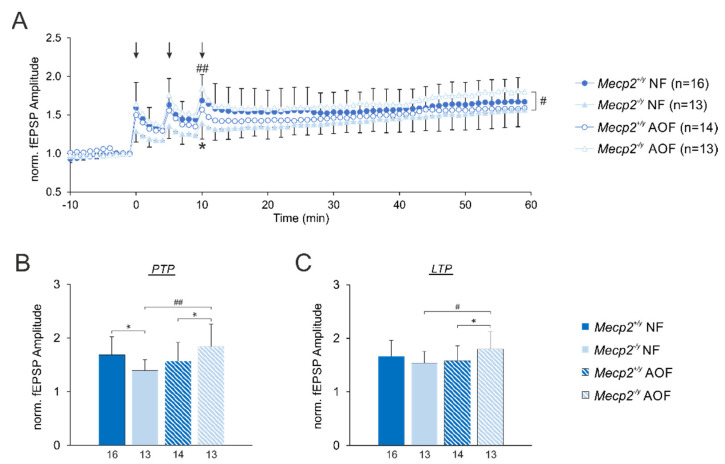
AO treatment improved synaptic plasticity in *Mecp2*^−/*y*^ mice. (**A**) The summary of the synaptic plasticity experiments depicts the normalized fEPSPs of the four male mouse groups. Arrows indicate the high-frequency stimulation (100 Hz, 1 s duration) applied to evoke short-term plasticity (post-tetanic stimulation (PTP)) as well as long-term plasticity (LTP). (**B**) The extent of PTP was quantified right after the 3rd stimulus train, and it was significantly reduced in *Mecp2*^−/*y*^ mice receiving normal food (NF) as compared with WT males. AO treatment fully reinstated PTP in *Mecp2*^−/*y*^ mice. The number of mice studied is indicated below each bar; shadings and patterns apply to panels B and C. (**C**) High-frequency stimulation induced stable LTP in all groups. After 60 min of recordings, LTP in *Mecp2*^−/*y*^ mice only tended to be slightly lower than that in WT males. Nevertheless, AO treatment significantly increased the extent of LTP in *Mecp2*^−/*y*^ mice. The significance of the PTP and LTP changes observed was assessed by two-way ANOVAs followed by all-pairwise Holm–Sidak *post-hoc* tests. Genotype-related differences are indicated by asterisks (* *p* < 0.05), and treatment-mediated differences are indicated by crosshatches (# *p* < 0.05, ## *p* < 0.01).

In WT males, high-frequency stimulation resulted in a clear potentiation of fEPSP amplitudes, which averaged 69% right after the 3rd stimulus train (PTP) and still measured 66% after 60 min (Figure 7). In contrast, in *Mecp2*^−/*y*^ mice the extent of PTP was significantly decreased; the extent of LTP tended to be less pronounced. In WT males, AO treatment did not noticeably affect PTP and LTP, but in *Mecp2*^−/*y*^ mice receiving AO food, both PTP and LTP became clearly more pronounced (Figure 7).

### 3.6. DNA Oxidation

**Figure 8:**
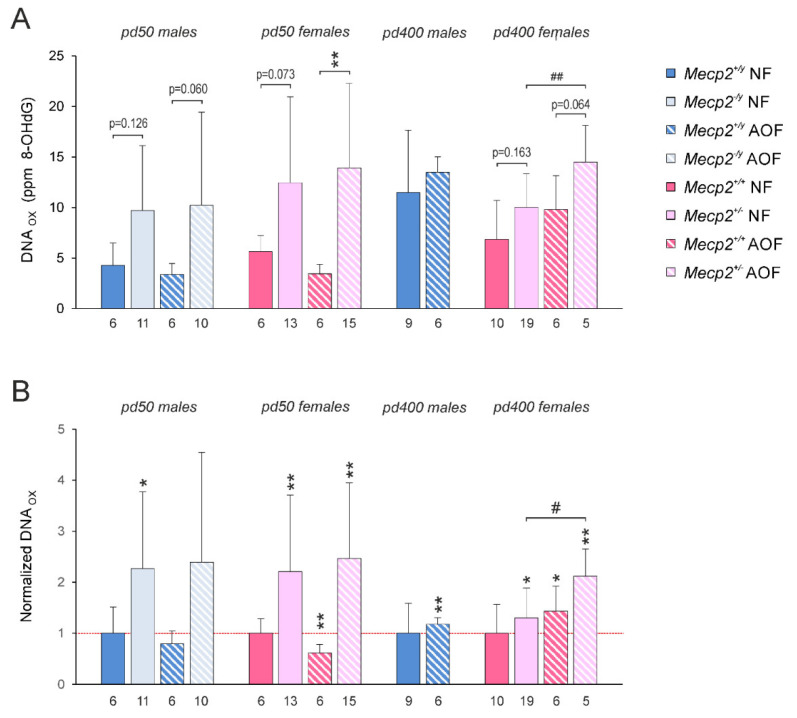
Intensified DNA oxidation in *Mecp2*-mutant cortex. (**A**) Both *Mecp2*^−/*y*^ and *Mecp2*^+/−^ cortices showed increased levels of DNA oxidation on pd 50. This trend also applied to *Mecp2*^+/−^ mice on pd 400. AOs only mediated some protection in WT mice but tended to mediate even higher levels of DNA oxidation in *Mecp2*-mutant mice. The number of mice studied is reported below each bar. Plotted are the averaged degrees of DNA oxidation, i.e., the detected amounts of 8-OHdG in parts per million (ppm). (**B**) In addition, the normalized changes as referred to the respective WT control group were calculated. Genotype-related significant differences are indicated by asterisks (* *p* < 0.05; ** *p* < 0.01), and AO-evoked changes among *Mecp2*^+/−^ mice are marked by crosshatches (# *p* < 0.05; ## *p* < 0.01). The statistical comparisons indicated in panel A were performed with two-way ANOVAs followed by all-pairwise Holm–Sidak testing. To rate the normalized DNA-oxidation levels (referred to respective WT controls defined as unity; see panel B), one-sample t-tests were used.

The oxidative stress in RTT results in the oxidative damage of proteins and lipids. Yet, it is largely unknown whether such damage also involves nucleic acids. Therefore, we quantified the oxidative DNA damage in male and female mice on pd 50 and on pd 400 and screened for the potential protective effects of AO treatment. For these analyses we chose the cerebral cortex, as it provides sufficiently large amounts of tissue. On pd 50, the cortical DNA oxidation, detected as 8-OHdG content, was comparable in male and female WT mice (Figure 8A). With aging (pd 400), higher degrees of DNA oxidation were detected, and WT males accumulated more oxidative damage than WT females. In *Mecp2*^−/*y*^ and *Mecp2*^+/−^ mice on pd 50 the extent of DNA oxidation was about doubled as compared with WT mice (Figure 8A). The normalization to WT control conditions emphasized these genotype-related differences. In addition, on pd 400, *Mecp2*^+/−^ mice showed a higher degree of DNA oxidation than WT females, but this difference was less pronounced than in younger females (Figure 8B). AO treatment slightly dampened the DNA oxidation in female WT mice on pd 50. It failed, however, to oppose the RTT-related intensified DNA oxidation in *Mecp2*^−/*y*^ and *Mecp2*^+/−^ mice (Figure 8). Instead, even a further increase in DNA oxidation became evident, which reached the level of significance in long-term AO-treated male and female mice on pd 400 (Figure 8B).

## 4. Discussion

### 4.1. Redox-Stabilizing Treatment Concepts

Oxidative stress contributes to the pathogenesis of RTT in patients as well as in various mouse models of this disorder [24,25,27,29,31,51]. The mechanistic source of this oxidative stress is an imbalance between the cellular ROS production by mitochondrial and non-mitochondrial mechanisms, and cellular ROS detoxification. This redox imbalance was shown to affect various cell compartments [24]. Accordingly, treatments dampening either cellular ROS production and/or boosting cellular redox buffering capacities were assessed as treatment concepts, and partial merits were confirmed.

The oral uptake of ω-3 polyunsaturated fatty acids decreased various oxidative stress markers in the blood samples of RTT patients and reduced the clinical severity by improving their motor functions, regularity of breathing, non-verbal communication, and myocardial systolic function [40,41]. In a first clinical trial, redox-stabilizing compound EPI-743 improved head growth as compared with the placebo-receiving group [42,52]. In female *Mecp2*^+/−^ mice, curcumin treatment ameliorated vascular function by dampening intravascular ROS generation, restoring the vascular expression of the endothelial nitric oxide synthase, and reinstating the availability of endothelial nitric oxide [36]. Moreover, N-acetylcysteine was shown to modulate oxidative stress by preventing extracellular glutamate release and excitotoxicity while increasing intracellular glutathione levels in the activated (LPS stimulation) cultured glia of *Mecp2*^−/*y*^ mice [53]. In addition, it improved the overall phenotypic appearance and behavioral performance (mobility, tremors, hindlimb-clasping, and respiration) in *Mecp2*^−/*y*^ mice. Optimum efficiency was, however, only obtained when N-acetylcysteine was not delivered as a free compound but rather conjugated with a dendrimer structure [53].

We previously demonstrated that the vitamin E derivative Trolox administered to acute or cultured *Mecp2*^−/*y*^ hippocampal tissue slices stabilized the cytosolic redox balance, dampened neuronal hyperexcitability, improved synaptic short-term and long-term plasticity, and increased hypoxia tolerance [23,38]. A systemic treatment of *Mecp2*^−/*y*^ mice with Trolox dampened lipid peroxidation and protein carbonylation within the cortex, partially restored the lowered blood glucose levels, and improved hippocampal hypoxia tolerance as well as synaptic short-term plasticity [37]. Moreover, Trolox ameliorated the exploratory behavior of *Mecp2*^−/*y*^ mice [37].

Here, we applied a therapeutic cocktail of AOs by combining N-acetyl cysteine, α-lipoic acid, and vitamin E, based on the rationale of taking advantage of potential synergistic effects. Furthermore, we chose a self-feeding oral administration of the AO diet, in order to prevent the negative impact of stress and handling effects, which were seen in our previous study with intraperitoneal injections of Trolox [37].

N-acetylcysteine itself has only poor radical scavenging capacity, but as a cysteine precursor, it promotes glutathione synthesis. Moreover, it is converted in mitochondria to sulfane sulfur species, which were proposed as the actual mediators of the N-acetylcysteine-mediated cytoprotection and antioxidant properties [54]. The dithiol compound α-lipoic acid is a potent mitochondrial cofactor and antioxidant; it chelates transition metals and stimulates cellular-glutathione synthesis [55,56]. Vitamin E derivatives efficiently scavenge hydroperoxyl radicals, thereby breaking lipid-peroxidation chain reactions. Moreover, they react with superoxide and singlet oxygen [57,58]. Each of these compounds permeate the blood–brain barrier [43].

In a mouse model of X-adrenoleukodystrophy, this triplet of AOs—at somewhat higher doses (850 mg/kg/day of N-acetylcysteine, 430 mg/kg/day of α-lipoic acid, 65 mg/kg/day of vitamin E)—reversed oxidative stress-mediated protein damage, prevented axonal degeneration, and improved locomotor functions [43]. Moreover, similar dietary supplementation with N-acetylcysteine, (500 mg/kg/day), α-lipoic acid (30 mg/kg/day), and α-tocopherol (15 mg/kg/day) dampened the oxidative stress and prevented the proinflammatory state that manifest in the aging rat brain [44].

### 4.2. Impact of AO-Feeding on Phenotypic and Systemic Parameters

In our RTT mouse model, the combined application of the three distinct AOs was meant to take advantage of their confirmed synergistic effects [43] and to assure a broad targeting of previously reported redox-defects in RTT. For instance, lowered blood-serum levels of vitamin E and reduced glutathione levels were confirmed in RTT patients [26,31], and reduced ascorbic acid and glutathione levels were proven in *post-mortem* brain tissue [59]. Moreover, the lowered activities of pivotal scavenging enzymes such as superoxide dismutase, glutathione peroxidase, and thioredoxin reductase were detected in RTT patients’ fibroblasts [32].

The AO cocktail was provided from the time point of weaning. This treatment successfully improved the general phenotypic appearance of *Mecp2*^−/*y*^ mice, as it stimulated their gain of weight, their growth, and their BMI. Hence, a beneficial impact on the distorted metabolic conditions in RTT [60,61] can be assumed. This indicates that the administered AOs did not only affect the central nervous system but also modulated the functions of peripheral organs. During long-term treatment, however, AO food led to a massive weight gain in female *Mecp2*^+/−^ mice, with adipose appearance, as compared with female WT and *Mecp2*^+/−^ mice receiving regular food. Unfortunately, long-term AO treatment could not be performed in male *Mecp2*^−/*y*^ mice due to their premature death. At present, therefore, it does remain unclear whether the massive weight gain in AO-treated *Mecp2*^+/−^ mice on pd 400 arises from the long-term treatment itself and/or may be linked to the heterozygous phenotype and its milder disease severity. For translational aspects this exact type of information would, however, be highly relevant.

Blood glucose levels were significantly reduced in *Mecp2*^−/*y*^ mice, which may have been due to the intensified carbohydrate metabolism detected in our recent metabolomics study on *Mecp2*^−/*y*^ cortex [61]. An amelioration of blood glucose contents in *Mecp2*^−/*y*^ mice was observed upon AO treatment. Instead, in *Mecp2*^+/−^ mice blood glucose levels were increased (or tended to be increased), which may have been due to their X-chromosomal mosaicism and less severe disease phenotype. These already elevated blood glucose levels markedly further rose in AO-fed *Mecp2*^+/−^ mice on pd 400, which may be linked to the above-mentioned obesity.

In contrast, the increased hematocrit in *Mecp2*^−/*y*^ and *Mecp2*^+/−^ mice was not rescued by AO therapy. This parameter obviously represents a systemic adaptation to intermittent systemic hypoxia [50], and since normal breathing was not reinstated with AOs, intermittent hypoxia can also be expected to persist.

The Kaplan–Meier survival curves demonstrated markedly shorter lifespans in *Mecp2*^−/*y*^ and *Mecp2*^+/−^ mice as compared with WT mice. A significant improvement caused by the administration of AOs was not evident in *Mecp2*-mutant mice. The previously reported trend of an even shortened life expectancy in male Mecp2-null mice intraperitoneally injected with N-acetylcysteine [53] was, however, not observed with our AO-cocktail-based therapy.

### 4.3. Partial Re-Instatement of Brain Morphometry

A promising finding is that some of the morphometric aspects associated with *Mecp2*-deficiency, such as reduced brain size and smaller neurons [62,63], were also ameliorated by AO treatment. This includes a solid trend towards the recovery of the reduced brain weight in AO-fed *Mecp2*^+/−^ females, larger cell-sizes of CA1 pyramidal neurons in male *Mecp2*-mutant mice as well as female *Mecp2*-mutant mice on pd 400, and a corresponding trend in female *Mecp2*-mutant mice on pd 50. Furthermore, an increased cortical-layer thickness was confirmed in AO-fed *Mecp2*^−/*y*^ mice. Hence, the brain structure itself can be modified successfully by an improved redox balance. In these aspects, the AO cocktail proved to be superior to the previously assessed Trolox treatment [37]. Likewise, in a first clinical trial, the redox-stabilizing compound EPI-743 was also found to improve the head circumference in RTT patients [52]. Despite partial reinstatement, the morphometric changes mediated by the AO cocktail were, however, not sufficient to also result in an improvement of behavioral performance.

### 4.4. Improvement of Synaptic Plasticity

The impairment of synaptic short-term and long-term plasticity is a hallmark of RTT and was reported in different facets in the brains of *Mecp2*-mutant mice [38,50,64,65]. Here, we only focused on male mice and the conditions of absolute *Mecp2*-deficiency. The studied cohort of mice showed a decreased extent of PTP. The changes in LTP were less pronounced here and were only evident as a tendency. Nevertheless, AO treatment proved to be efficient in reinstating synaptic short-term plasticity (PTP) and improving the extent of LTP in *Mecp2*^−/*y*^ mice. Any adverse side effects of the AO cocktail on synaptic functions were not found. This AO-mediated stabilization of hippocampal synaptic plasticity is in line with our previous observations of the administration of the vitamin E derivative Trolox, which was assessed in vitro as well as in vivo [37,38]. Hence, our findings obtained at the CA3–CA1 synapse further support the concept that an improved cellular redox balance contributes to an improved synaptic plasticity in *Mecp2*-deficient hippocampus. Nevertheless, the achieved synaptic stabilization seemed not to be sufficient to translate into improved behavioral parameters. Hence, it remains to be clarified what benefit can also be mediated by the AO cocktail on the synaptic functions of other brain regions.

### 4.5. DNA Oxidation in Mecp2-Mutant Cortex

So far, oxidative damage to especially proteins and lipids was addressed in RTT [27,33,66]. Information on oxidative DNA damage—even though it is expected [67]—is still sparse. Therefore, we quantified the levels of 8-hydroxy-2‘-deoxyguanosine (8-OHdG) as a marker of the extent of DNA oxidation and confirmed for the first time that oxidative DNA damage did occur in the cortex of *Mecp2*^−/*y*^ and *Mecp2*^+/−^ mice. These results on an RTT-related DNA oxidation are remarkable, because not only severely symptomatic *Mecp2*^−/*y*^ mice but also heterozygous female *Mecp2*^+/−^ mice were already affected on pd 50, i.e., at an age they were still considered to be largely presymptomatic. As oxidative changes in methyl-CpG sites within the DNA interfere with proper MeCP2 binding [68], increased DNA oxidation may thus further aggravate the already disturbed gene-regulation in RTT. AO treatment only dampened the DNA oxidation in female WT mice on pd 50, but it failed to ameliorate the conditions in *Mecp2*-mutant mice at that age. With long-term treatment, the extent of DNA oxidation in AO-fed WT and *Mecp2*^+/−^ females increased even further, which again emphasizes that chronic AO supplementation for extended periods does not only lose its efficacy but may even turn into unexpected opposite effects and aggravate symptoms.

### 4.6. Behavioral Performance and Regularity of Breathing Were Not Ameliorated

Detailed behavioral tests confirmed the expected RTT-related disturbances in motor functions with reduced general activity and exploratory behavior in male and female *Mecp2*-mutant mice [14,45]. Despite the confirmed stabilization of synaptic plasticity at the hippocampal CA3–CA1 synapse of male *Mecp2*-mutant mice, any improvements of the behavioral parameters could not be confirmed upon AO treatment. Instead, *Mecp2*^+/−^ mice showed a further decline in motor functions with continued AO treatment on pd 200, which may have been due to their weight gain and obese phenotype. Furthermore, an age-related decline in female WT mice during rotarod testing became evident.

Plethysmography revealed the characteristic irregular breathing in male and female *Mecp2*-mutant mice [69,70,71]. Combining unrestrained whole-body plethysmography with video-recordings revealed that—besides the frequent apneas—other major sources of the altered breathing pattern had to be considered in *Mecp2*^−/*y*^ and *Mecp2*^+/−^ mice. These included the reduced exploratory behavior and the less intense grooming, both of which resulted in a shift in the respiratory frequency composition towards lower frequencies. An improvement of the regularity of respiration could not be seen upon AO treatment in *Mecp2*^−/*y*^ or *Mecp2*^+/−^ mice. This corroborates our previous observations that the disturbed breathing of *Mecp2*^−/*y*^ mice persisted despite Trolox treatment [37]. In contrast, in RTT patients, the respiratory function improved upon oral ω-3 PUFA uptake [40]. EPI-743, a compound stabilizing redox balance and cellular-glutathione content, did not, however, improve the regularity of breathing in RTT patients [42,52].

Thus, further research is needed to clarify why the AO-mediated partial improvements in brain morphometry and synaptic plasticity in *Mecp2*-mutant mice did not readily translate into systemic, behavioral, and breathing improvements. This could involve the performance rating of the different neuronal networks with, e.g., more detailed electrophysiological analyses. Redox imaging at the cellular level in brain tissue slices would be another valuable approach to rate cellular redox homeostasis in general during RTT pathogenesis and to clarify potential AO-mediated improvements in different brain regions.

## 5. Conclusions

In conclusion, only some of the complex features that characterize the RTT-related phenotype in *Mecp2*-mutant mice were ameliorated by the AO cocktail. This partial protection was evident under the severe disease conditions presented by hemizygous male *Mecp2*^−/*y*^ mice as well as under the milder disease conditions manifesting in heterozygous *Mecp2*^+/−^ females, which represented the clinically relevant X-chromosomal mosaicism of the RTT patients. Other prominent RTT features, such as distorted breathing or behavioral deficits, did not, however, improve. It also became evident that the therapeutic potential is limited in time, as with chronic life-long AO treatment of *Mecp2*^+/−^ mice, the protection faded or the conditions became even worse for some of the parameters studied. Hence, for continuous treatment, well-controlled adjustments of AO dosages to the stages and the severity of RTT seem essential. As disease severity is strongly determined by the hemizygous and heterozygous genotype, gender-specific AO-dosing concepts may also be required. With the majority of patients being females, this latter aspect would of course be more relevant for further mouse studies than for clinical application. In healthy subjects, e.g., WT mice, the existence of a redox optimum can be assumed. Distorting this optimum by the unnecessary consumption of AOs or redox-stabilizing compounds, i.e., by chronic “over-buffering”, obviously results in multi-faceted disturbances. This may also apply to the overdosing of AOs or their gradual accumulation during long-term treatment under disease conditions. It should be considered, however, that in patients, the dosing and administration of therapeutic compounds can be controlled and adjusted more reliably than in mice.

In addition to some morphological aspects of brain structure, hippocampal synaptic plasticity also improved upon AO-cocktail treatment as did the systemic parameters (growth). Hence, quite a spectrum of effects was mediated by the combined AOs. Therefore, it would be of interest to clarify in future studies the detailed benefits of the AO cocktail not only for the spectrum of mitochondrial functions but also for the complex metabolic alterations and the performance of neuronal networks in RTT. The gender dichotomy of the AO-mediated effects is another aspect to be clarified. In addition, in terms of systemic and general phenotypic parameters, it would be highly relevant to determine which effects are mediated by AOs on peripheral organs and which particular changes are evoked in the central nervous system. This shall reveal further crucial insights into the full pharmacotherapeutic potential of AOs, but also the associated limitations for the treatment of RTT-related symptoms.

## Figures and Tables

**Table 1:** Phenotypic parameters distinguishing WT and *Mecp2*-mutant mice and their modulation via antioxidant food (AOF). Listed data are means ± standard deviations (SDs); *n* refers to the number of mice studied. Significant genotype-related differences among WT and *Mecp2*-mutant mice are indicated by asterisks (* *p* < 0.05, ** *p* < 0.01). Those significant differences among matching genotypes that arose from AO treatment are identified by crosshatches (# *p* < 0.05, ## *p* < 0.01).

Genotype	Phenotypic Parameters				
	Body Size (cm)	Body Mass Index (g/cm^2^)	Hematocrit (%)	Blood Glucose Level (mg/dl)	*n*
*Mecp2*^+/*y*^ NF (pd 50)	8.27 ± 0.34	0.3195 ± 0.0289	44.61 ± 1.99	244.73 ± 40.33	40
*Mecp2*^−/*y*^ NF (pd 50)	6.91 ± 0.57 **	0.2599 ± 0.0533 **	49.56 ± 3.73 **	179.88 ± 52.90 **	43
*Mecp2*^+/*y*^ AOF (pd 50)	8.39 ± 0.46	0.3126 ± 0.0291	44.72 ± 2.80	238.93 ± 41.33	41
*Mecp2*^−/*y*^ AOF (pd 50)	7.53 ± 0.52 **, ##	0.2863 ± 0.0360 **, ##	49.71 ± 3.32 **	197.26 ± 53.37 **	43
*Mecp2*^+/+^ NF (pd 50)	7.95 ± 0.38	0.2816 ± 0.0239	44.94 ± 1.95	207.80 ± 35.36	41
*Mecp2*^+/−^ NF (pd 50)	8.00 ± 1.45	0.2720 ± 0.0424	46.43 ± 2.52 *	233.53 ± 50.96 *	43
*Mecp2*^+/+^ AOF (pd 50)	7.98 ± 0.25	0.2779 ± 0.0259	45.67 ± 1.86	223.38 ± 38.79	42
*Mecp2*^+/−^ AOF (pd 50)	7.76 ± 0.42	0.2869 ± 0.0267 #	47.61 ± 2.64 **, #	229.60 ± 34.98	40
*Mecp2*^+*/*+^ NF (pd 200)	8.60 ± 0.22	0.3487 ± 0.0460	44.48 ± 2.07	220.20 ± 31.47	25
*Mecp2*^+/−^ NF (pd 200)	8.53 ± 0.32	0.3887 ± 0.0803 *	45.98 ± 1.39 *	228.67 ± 29.42	18
*Mecp2*^+/+^ AOF (pd 200)	8.84 ± 0.37	0.3311 ± 0.0176	44.13 ± 1.92	213.38 ± 23.58	8
*Mecp2*^+/−^ AOF (pd 200)	8.63 ± 0.26	0.4107 ± 0.0577 *	46.83 ± 1.90 *	227.83 ± 38.01	6
*Mecp2*^+/+^ NF (pd 400)	9.06 ± 0.36	0.3859 ± 0.0537	42.46 ± 2.60	218.39 ± 31.27	38
*Mecp2*^+/−^ NF (pd 400)	8.86 ± 0.62	0.4708 ± 0.1286 *	45.09 ± 3.65 **	223.33 ± 29.82	21
*Mecp2*^+/+^ AOF (pd 400)	9.15 ± 0.38	0.4042 ± 0.0615	43.77 ± 2.08	214.05 ± 37.96	20
*Mecp2*^+/−^ AOF (pd 400)	8.86 ± 0.38	0.6472 ± 0.1025 **, ##	46.86 ± 2.09 **	267.60 ± 25.41 **, ##	5

**Table 2:** Brain morphometric parameters distinguishing WT and *Mecp2*-mutant mice and their modulation via AO treatment. Analyses were performed on PFA-fixed brains. Cell and layer structures were visualized with Nissl staining. Listed data are means ± SDs; *n* reports the number of mice Scheme 0. * *p* < 0.05, ** *p* < 0.01), and AO-treatment-mediated changes are marked by crosshatches (# *p* < 0.05).

Genotype	Morphometric Parameters				
	Hemisphere Size (mm^2^)	Thickness CA1 *Str. Pyramidale* (µm)	Thickness CA3 *Str. Pyramidale* (µm)	Thickness DG *Str. Granulosum* (µm)	*n*
*Mecp2*^+/*y*^ NF (pd 50)	27.03 ± 1.06	60.7 ± 3.15	71.00 ± 5.81	79.10 ± 2.19	6
*Mecp2*^−/*y*^ NF (pd 50)	23.91 ± 0.37 **	56.18 ± 6.00	67.79 ± 5.24	77.09 ± 7.52	6
*Mecp2*^+/*y*^ AOF (pd 50)	26.69 ± 0.68	58.57 ± 2.63	71.05 ± 3.68	79.54 ± 2.47	7
*Mecp2*^−/*y*^ AOF (pd 50)	23.68 ± 0.86 **	56.62 ± 3.60	66.82 ± 3.43	75.96 ± 5.38	7
*Mecp2*^+/+^ NF (pd 50)	26.92 ± 1.41	61.86 ± 2.89	74.84 ± 2.15	80.66 ± 2.72	6
*Mecp2*^+/−^ NF (pd 50)	25.06 ± 0.65 **	56.42 ± 4.62 *	67.94 ± 2.91 *	78.31 ± 4.23	6
*Mecp2*^+/+^ AOF (pd 50)	27.64 ± 0.74	65.06 ± 3.87	68.50 ± 4.39 #	80.64 ± 6.12	6
*Mecp2*^+/−^ AOF (pd 50)	25.94 ± 1.02 **	58.54 ± 4.62 **	71.00 ± 6.02	80.50 ± 4.73	7
*Mecp2*^+/+^ NF (pd 400)	27.16 ± 0.67	55.08 ± 2.46	66.37 ± 4.03	76.31 ± 4.05	7
*Mecp2*^+/−^ NF (pd 400)	22.97 ± 1.36 **	50.84 ± 2.09 **	59.96 ± 3.32 *	72.90 ± 3.30	8
*Mecp2*^+/+^ AOF (pd 400)	26.82 ± 0.94	53.02 ± 2.43	62.35 ± 7.25	74.01 ± 4.08	7
*Mecp2*^+/−^ AOF (pd 400)	23.05 ± 1.63 **	52.65 ± 0.64	60.61 ± 3.69	74.79 ± 3.68	6

## Data Availability

Data is contained within the article.
